# Pride Before a Fall: Shame, Diagnostic Crossover, and Eating Disorders

**DOI:** 10.1007/s11673-019-09923-3

**Published:** 2019-07-01

**Authors:** Rose Mortimer

**Affiliations:** Department of Psychiatry, Warneford Hospital, University of Oxford, Warneford Lane, Oxford, OX3 7JX England

**Keywords:** Eating disorder, Shame, Bulimia, Anorexia, Diagnostic labels

## Abstract

This paper discusses the findings of qualitative research that examined the accounts of five “mostly recovered” ex-patients who had experienced transition between two or more eating disorder diagnoses. This study found that, in the minds of participants, the different diagnostic labels were associated with various good or bad character traits. This contributed to the belief in a diagnostic hierarchy, whereby individuals diagnosed with anorexia nervosa were viewed as morally better than those diagnosed with bulimia nervosa or binge eating disorder. Consequently, diagnostic crossover from a “better” to a “worse” eating disorder was often experienced as shameful moral failing, and a new diagnosis impacted the individual’s sense of self-identity. These findings are of significance for both ethicists and clinicians; the paper concludes by outlining the relevance and possible clinical implications of shame in diagnostic crossover and suggesting avenues for future research.

## Introduction

This paper explores the phenomenology of diagnostic crossover in eating disorders (EDs), focusing specifically on the first-person accounts of five “mostly recovered” ex-patients. Diagnostic crossover occurs when an individual moves from one ED diagnosis to another.

The paper begins by outlining the various diagnostic categories of EDs within the fifth edition of the *Diagnostic and Statistical Manual for Mental Disorders* (DSM-5), focusing specifically upon anorexia nervosa (AN), bulimia nervosa (BN), and binge eating disorder (BED). Previous research concerning the nature and prevalence of diagnostic crossover is then discussed, followed by an overview of the literature exploring the association between EDs, self-identity, and moral virtue. After discussing the study methodology, the paper then presents the first-person accounts of participants, followed by a discussion of the key findings focusing particularly upon shame. The paper concludes by outlining possible clinical implications of shame in diagnostic crossover and suggesting avenues for future research.

## Background

### Diagnosis and Diagnostic Crossover

DSM-5 lists eight categories of feeding and eating disorder, and this paper focuses on three: anorexia nervosa (AN), bulimia nervosa (BN), and binge eating disorder (BED). AN can also be separated into two sub-types: anorexia-restrictive type (ANR) and anorexia-binge-purge type (ANBP). An individual will not be given more than one diagnosis at a time, however, there are many similarities between the ED diagnostic categories in DSM-5. Notably, several diagnostic criteria are shared by more than one disorder. For example, binge eating is common to the diagnostic criteria of ANBP, BN, and BED, and self-evaluation is unduly influenced by body shape and weight in both AN and BN (American Psychiatric Association 2013). In this sense, it is the distinct *combination* of symptoms or behaviours that determines the diagnosis, and acquiring or losing one symptom can result in transition to a new diagnostic category, a process known as diagnostic crossover.

An individual’s experiences or behaviours need not change dramatically to warrant a different diagnostic label; for example, a patient with ANBP who gains sufficient weight will transition to BN, and vice versa. Given that weight and symptoms tend to fluctuate regularly, and that diagnosis is made on the basis of positive symptoms over just three months, some individuals may experience recurrent diagnostic crossover over time (Milos et al. 2005).

Research suggests that diagnostic crossover in EDs is relatively common; one study found that approximately 34 per cent of individuals with an initial diagnosis of AN later develop BN, and 14 per cent of those originally diagnosed with BN go on to develop AN (Eddy et al. 2008). Crossover between the AN subtypes is believed to be even more common, occurring in 44–60 per cent of cases (Eddy et al. 2008; Eddy et al. 2002).

### Eating Disorders and Moral Character

Since the 1960s and 70s it has been well established in the literature that EDs are closely intertwined with moral values, both in the minds of those diagnosed and within society (Bruch 1973; Duker and Slade 2003). As Giodano writes: “at the basis of the admiration for a thin body or an empty body there are moral values: self-control, perfectionism, purity, intellectual achievement, will power and hard work, a cluster of values with a long history” (2005, 156). Research has also shown how BN and BED are typically viewed in opposition to AN, and therefore become symbolic of moral weakness. Indeed, despite significant similarities between the various different EDs, they are often conceived of in binary terms: chaos and control; disgust and purity; vice and virtue (see for example: Saguy and Gruys 2010; Eli 2018; Squire 2003). These virtues and vices are gendered, often focused on ideal notions of specifically *feminine* morality (Burns 2004; Giordano 2005, 157–158).

Additionally, it is well known that EDs strongly impact self-identity. This is especially likely in the case of ego-syntonic disorders such as AN, when patients sometimes struggle to identify an “authentic self” that is separate from the disorder (Hope et al. 2011; Marzola et al. 2015). Consequently, individuals with AN may incorporate symptoms within their self-concept and ideal identity, and pursuing the disorder comes to represent the pursuit of personal values (Mulkerrin, Bamford, and Serpell 2016). In this sense ED identity and the moral values it entails can become sources of pride and of shame (Skårderud 2007).

### The Rationale for This Study

A phenomenological study of diagnostic crossover is of relevance to both clinical practice and academic bioethics.

Bioethics is inherently concerned with ethical issues arising in the life sciences, particularly medicine. Therefore, this research is of relevance to bioethics in that it brings to light and investigates the moral dimensions of psychiatric diagnosis. Philosophers such as Fulford (2005) and Sadler (2002) have examined how psychiatric diagnostic classifications are influenced by the values held by mental health professionals and the society in which they practice. The present study contributes to this literature by exploring ways in which a specific group of five ex-patients understand and experience the interplay of moral value and psychiatric diagnosis, and how this impacts their self-understanding and moral identity.

Research of this kind may also have implications for clinical practice. Though the present research involved a small sample and did not seek to create generalizable findings, this study lays the groundwork for future research exploring the experience of patients who have undergone diagnostic crossover. These insights into patient experience could contribute to discussions about the categorization of diagnostic criteria in future editions of the DSM and may also lead to the development of treatment that is sensitive to the first-person experience of diagnostic crossover and its impact on individual identity. Further, such research may help clinicians and others become more aware of the barriers to disclosure of diagnostic crossover, and to put in place strategies for identifying vulnerable patients and providing support that is sensitive to the moral dimensions of the experience.

## Methods

### Summary

Semi-structured interviews were conducted with eight young women, all of whom had experienced at least one ED diagnosis from a medical professional in the last ten years, but who now consider themselves “mostly recovered.” Although the study had been initially designed to explore participants’ accounts of recovery, in the process of conducting interviews the theme of diagnostic crossover arose and was identified as important. This paper focuses specifically on the accounts of the five “ex-patients” who had undergone diagnostic crossover, reflecting on what this experience meant for their understanding of self-identity and recovery. All five of these participants had experienced crossover from AN to BN and/or BED, but not in the other direction. The results from the larger study, which considered recovery more broadly and which did not focus solely on diagnostic crossover, are discussed elsewhere (Mortimer 2015).

### Interpretative Phenomenological Analysis

This study was conducted using interpretative phenomenological analysis (IPA), a phenomenologically informed method for qualitative research. IPA seeks to understand “how people make sense of events, relationships, and processes in the context of their particular lifeworlds” (Larkin, Eatough, and Osborn 2011, 330). The aim is not to create an “objective” or generalizable account of an event or state, but rather to understand how the event was experienced from the first-person perspective of specific individuals.

IPA draws from hermeneutic phenomenologists such as Heidegger (1927) and Merleau-Ponty (1962), emphasising the fact that cognition is always embodied and situated in context; all understanding is an interpretation from a particular perspective within the social world. We are, Heidegger claims, beings-in-the-world, and so meaning is inseparable from the shared lived world and the physical bodies that inhabit it. Therefore, whilst IPA focuses upon the first-person experiences of individuals, it also strives to situate these within context and to consider the relevant social and cultural factors at play.

IPA is appropriate for the present study since—given the small sample size—the aim was not to make “objective” claims about the validity of diagnostic categories or to draw generalizable conclusions from participants’ narratives. Rather, this study explores in detail the perspectives of individuals upon a shared experience and seeks to make sense of the meanings they ascribe to diagnostic crossover. Like most IPA studies, this research takes an idiographic approach, involving a relatively small number of participants so that the narrative of each can be explored in detail; to this end, some relatively long quotations are included in this paper. The aim is to balance the requirement to give voice to the concerns of participants with the aim to make sense of these narratives through analysis (Larkin, Watts, and Clifton 2006).

### Sampling and Eligibility Criteria

In order to participate in this research, participants had to fulfil each of the following eligibility criteria:

1. Is over 18 years of age
2. Has experienced at least one ED diagnosis from a medical practitioner in the last ten years
3. Considers him or herself to be “mostly recovered” or “recovered” from the ED(s)

Participants were not provided with any further guidance as to what might constitute “mostly recovered,” since the goal of the original study was, in part, to examine this very question: what do participants consider “recovery” to be or mean? Focusing on participants’ own assessment of their recovery status is appropriate for IPA, since it seeks a subjective rather than an objective account of this complex concept and recognises that a purely “biomedical model” is insufficiently nuanced to explore the richness of participants’ experiences. During interviews it became apparent that the participants’ present-day experience of ED thoughts, beliefs, or behaviours varied greatly, yet each considered herself at least “mostly recovered.”

### Recruitment

Participants were initially identified and contacted through the social networking website Facebook. These individuals had all received treatment at a specific National Health Service ED inpatient service in the south east of England within the last ten years. A snowballing strategy was then used whereby participants recommended others who might wish to be involved in the study.

This report is part of a larger study focusing on ED recovery, which has a total sample of eight. However, for this paper only the accounts of participants who had undergone crossover were included in the analysis. All five participants whose responses are included in this paper had experienced crossover from AN to BN and/or BED. Further information about these participants in included in Table 1.

**Table 1:** Participant details

Name	Age	Age at onset of ED	Recovered?	ED Diagnoses (most recent first) *=self-diagnosed
Ruth	22	13	Mostly	BN*; ANR
Hollie	22	16	Mostly	BN; AN*
Marianne	21	12	Mostly	BED*; ANR
Natalie	21	12	Mostly	BN; BED*; ANR
Sarah	26	12	Mostly	BN; ANR

### Data Collection and Analysis

Interviews were semi-structured and lasted between forty-five minutes and two hours. Interviews followed a topic guide that was designed to focus on three distinct but related areas: recovery, diagnostic categories, and identity.

Analysis was iterative and inductive. The analysis began with free coding of the transcripts, followed by detailed line-by-line coding. Coding was conducted using the constant comparison method, whereby each transcript is initially coded then revisited in the light of subsequent interviews so as to develop themes and codes that can be constantly revised and compared (Glaser 1965).

### Ethical Considerations

Ethics approval for this study was provided by King’s College London’s Research Ethics Committee, reference number: HR-14/15-0456.

All participants were given an information sheet, and all provided informed consent. All interview transcripts were anonymized, and all names used in this paper are pseudonyms.

In order to participate in this research, participants had to be of a self-reported healthy weight and consider themselves “recovered” or “mostly recovered” from their ED. Although EDs can affect people at any weight, these criteria were designed in part to exclude the most vulnerable participants and to minimize the risk of distress or “triggering” during interviews.

## Findings

A key finding from participant interviews was the experience of shame as a consequence of diagnostic crossover. Participants described the existence of a diagnostic hierarchy whereby certain EDs, and the individuals diagnosed with them, were perceived as “better” than others. Exploring what “better” means in this context led to a careful analysis of how character, self-image, and moral identity are experienced by individuals who undergo diagnostic crossover. In the following discussion, these findings are divided into three sections:

- Better diagnosis, better person
- Reading moral character through the body
- Diagnostic crossover: identity in flux

The first section explores some of the ways in which participants viewed moral character as intertwined with diagnostic categories or labels and therefore experienced EDs as existing within a hierarchy of better and worse people. The second section examines how moral character is often “read” from one’s physical appearance and therefore explores the ways in which participants experienced moral identity in relation to the body. The final section explicitly considers participants’ experiences of diagnostic crossover and how the movement from one ED to another was experienced as an “identity crisis” and shameful moral failing. The discussion that follows explores shame in more detail by drawing on relevant philosophical literature.

### Better diagnosis, better person

Anorexia is like the top one, you’re an exemplar of resilience and determination and you work hard, you don’t indulge, it’s incredibly puritanical […] I’m not really sure that I would ever have referred to myself as having anorexia as I didn’t feel [pause] I wasn’t good enough for that. [Hollie]

Hollie clearly identified AN as the best or even most desirable ED in a diagnostic hierarchy. For Hollie, those diagnosed with AN are “exemplar[s]” of a number of admirable character traits such as resilience and determination. Her description of herself as not “good enough” seemingly denotes both a moral standard and a skill set; she views herself as insufficiently good at restricting her food intake to qualify as “anorexic,” and this failure in part stems from the fact that she is not a good enough person and lacks the character traits necessary to successfully “work hard” to resist food.

Similarly, Natalie describes a hierarchy of ED diagnoses, suggesting that individuals with a “better” diagnosis, such as AN, are worth more than those with a “worse” diagnosis, like BN or BED:

There is the perception that AN is this extreme manifestation of will power, [and] will power is a positive quality, and hence you are from the outset imbuing that diagnosis with this strength, and therefore bingeing and bulimia are chaos, that’s negative, so from the very start that these labels are tied up with value judgments, […] I attach value to the different labels, like I think I was worth more as a person when I was anorexic than when I’m bulimic, I sort of think of it as a very hierarchical thing. [Natalie]

Whilst all participants identified “better” and “worse” EDs, associating these with better and worse character traits, Hollie explicitly uses moral language to suggest that the diagnostic hierarchy is grounded in judgments about moral character and virtue.

I thought there was something morally good about restricting. I thought it was piggish and disgusting that someone could eat lots like chocolate and not have control over their impulses. […] I can remember turning round to my family who are über-Catholic, and they’re also greedy, and I said with so much venom, “greed is also a sin, you think you’re a good Catholic but you’re not because you are greedy and that’s something I will not be.” [Hollie]

Hollie suggests that uncontrolled overeating is *morally* bad rather than just unhealthy, and therefore by restricting she guards against the moral stain of “greediness.” Indeed, she views herself as morally superior because of her ability to restrict her food intake and implies that she is an overall better kind of person than her family members who cannot always control their impulse for chocolate.

Importantly, participants suggested that the character traits associated with various diagnostic labels were not exercised only in relation to eating or weight control but extended into other areas of their lives, particularly their academic work.

If you have an ED that’s mainly about restricting, then it’s about perfectionism, so that’s normal here [at university], because we are all perfectionists, we like having good grades and working hard, whereas bingeing and purging doesn’t fit that because it’s a failure as opposed to something you’re proud of […], so now, yeah, it’s more a feeling of shame, like I’m not good enough to belong here. [Sarah]

I associated my thinness with success because when I was doing my GCSEs and I did really well, I was very thin […]. Anorexia requires this extreme resilience and determination and, well, it’s an ideal that is actually hammered home in school, you’re told to be diligent, and in work, the people who work the hardest do the best. [Hollie]

Sarah suggests that it is “normal” for university students to display traits like “perfectionism” which are associated with AN. Indeed, Hollie explains that such diligence is taught in schools as a way of encouraging academic success. In contrast, the BN identity entails character traits that make Sarah feel she doesn’t “belong” in a university environment, because she isn’t “good enough.” Here, the word “good” seemingly indicates both an academic and a moral standard to be met.

### Reading Moral Character Through the Body

Central to the diagnostic hierarchy is the differentiation between EDs that involve low body weight—AN and its subtypes—and those that do not. Participants often described BN and BED as more shameful in part due to the physically larger body associated with this diagnosis, and expressed concern about the ways that others would form judgments about their character from their physical appearance.

Anorexia—they’re the sad skinny girls, but internally they’re … well you feel really quite strong, you’re able to despite everything, despite people shouting and crying, you continue to do this one thing. Bulimics are less in trouble and that’s the sad thing because if you have an ED that makes you thin, oh tragedy but oh interest. But if you have an ED that makes you fat, then you’re no different to the fifty-year-old women who use Weight Watchers and can’t do anything about their proclivity for cheese. You know? It’s just conflated with general laziness or being unaware or not caring. [Marianne]

Marianne focuses on the physical body in order to clearly differentiate between the “skinny girls” with AN and “eating disorders which make you fat.” Because the bulimic body is often physically unremarkable, Marianne describes BN as being in some ways indistinguishable from “normal” overeating. This image is presented as mundane, in contrast to Marianne’s portrayal of AN as interesting and tragic. By blurring the boundary between “eating disorders which make you fat” and “normal” overeating, Marianne, like Hollie, emphasizes the idea that BN is associated with a shameful flawed character. She suggests that any weight gain resulting from this disorder is seen to be the result of “general laziness” rather than pathology.

For Marianne, the character traits associated with AN are not only more admirable but also more unique and special. She implies that many people are overweight, and as such unawareness or laziness is not only morally bad but also commonplace and unremarkable. Yet the extreme willpower associated with AN is seen to mark the anorexic person out as special, and the physically emaciated body much more notable than the large body in a society where so many people are overweight.

For Ruth, weight gain as a result of transition to BN was experienced as shameful because she feared that other people would wrongly attribute negative character traits to her on the basis of her physical appearance.

It was becoming fat that felt so alien. […] in my head I’m a thin, small person. I’m organised, I’m controlling, I’m pretty neurotic actually [laughs] but I’m really, really aware of what I eat, you know? But I see this fat, lazy person in the mirror, and it is really shocking because like people will think I just don’t care, but I do, it’s like my body doesn’t reflect what’s going on in my head, and I’m so ashamed when I think about how, how others see me, what they think. [Ruth]

The misalignment of lived and biological body in AN is well documented; physically the body is light and emaciated, yet the person often experiences their body as cumbersome and fat (Keizer et al. 2013). As a result, the individual feels alienated from the body and seeks to control it—and through the body, to control her identity—by starvation (Svenaeus 2013). However, Ruth is describing is something different; diagnostic crossover from AN to BN entails weight gain, and she feels as though her now physically larger body misrepresents her character or personality. Ruth holds onto the character traits that she associates with AN and which she values—good organisation skills, being “controlling”—and a heightened awareness around what she eats. But she worries that those who see her physically larger body will infer that she overeats and will assume that she is “lazy” and doesn’t “care.” Thus, although Ruth discusses the physical symptoms of BN, she is centrally concerned with the kinds of personality traits that she feels symptoms such as weight gain represent or imply.

### Diagnostic Crossover: Identity in Flux

The above has described participants’ accounts of a diagnostic hierarchy, rooted in the close association of ED diagnosis and moral character. Consequently, movement between diagnostic categories was experienced by participants as an “identity crisis” since they no longer knew how to understand their own moral character.

I think the transition to binge eating was terrifying […] because of the extent to which I felt like I didn’t recognise myself; it was like, “I don’t know what I’m doing”; I felt like I became a different person. [Natalie]

And I was so ashamed of what I’d become. Being anorexic was always a badge of honour when I was young, whereas being bulimic is a source of total shame and humiliation, especially after having been anorexic. I didn’t know you could be bulimic after anorexia, and I didn’t know what I had was bulimia, so I felt like I was the only person in the world this had happened to, like the worst anorexic ever. I didn’t know what to call myself. Just a fat girl with no will power. [Ruth]

These accounts clearly describe the sense of not recognizing oneself or one’s own behaviour. Ruth depicts BN in terms of its contrast with AN, simultaneously clinging to the AN identity —“the worst anorexic ever”—whilst accepting that this prior identity no longer fits her behaviours and appearance.

Similarly, Marianne discusses diagnostic crossover from the perspective of her former “anorexic” self:

… it’s weird to imagine your twelve or thirteen-year-old self allowing [diagnostic crossover] to happen. She’d be horrified. Absolutely. […] Who I was back then, would, would be horrified, and there was so much pride attached to the strength of that […] it still makes me feel quite ashamed, I think. [Marianne]

Marianne talks of her past self as a different person—“who I was back then”—and imagines the censure of the previous disciplined self. BN is shameful not just because of the bad character traits associated with it, but because it denotes a “fall from grace” whereby one loses the exemplary characteristics embodied by one’s former self.

Sarah powerfully describes this same experience:

Every moment that you’re not eating when someone else is, you’re taking pride from that moment. Even if everything else is going wrong, you still get to be the skinny one, it’s a safety plan for your self-esteem, constant accomplishment […] But then bingeing and purging, there isn’t a positive to it […] and you have to be alone, I think mainly the having to be alone part. You can’t really confide to anyone about it, or if you do you really have to risk that they will look at you and think you throw up and that’s disgusting. It’s a lack of understanding. I think a lot of people understand where anorexia comes from, it’s just an extreme diet, whereas this [BN] isn’t, it’s a lack of self-control. But it’s not like just saying, “Oh well stop, just have more self-control,” that doesn’t work, you’ve told yourself that many times. […] I was just very ashamed of putting on weight, ashamed of eating food, and also quite lonely. So, yeah, it was a bit miserable, and it just transformed completely from anorexia to bulimia. [Sarah]

Sarah describes the shame that accompanies “transformation” from AN to BN, and the overwhelming feelings of loss; loss of the self-control that used to be a source of pride and self-worth and the loss of friends that comes with increasing shame and isolation. Whilst restricting food can be done in the company of others, bingeing and purging must be done alone and in secret and then cannot be discussed for fear of judgment.

The accounts of participants presented here poignantly illustrate the impact of diagnostic crossover on moral identity. Since the character traits associated with AN are so closely tied to an individual’s sense of self-worth, diagnostic crossover results both in self-loathing and a certainty that others will also view one as worthy only of contempt and disgust. This leads to intense feelings of shame.

## Discussion

### Shame as a Moral Emotion

For participants in this study, diagnostic crossover was a shameful experience. Although they described shameful behaviours and bodily shame, they often also spoke of the shameful character traits that they felt caused and were revealed through their body size or behaviour. Participants associated AN with virtues such as self-control, strength, diligence, resilience, perfectionism, and hard work, whereas BN and BED were associated with morally bad character traits (or vices) such as laziness, greed, weakness of will, and lack of self-discipline. Therefore, crossover from AN to BN or BED was experienced as moral failing.

There is a significant philosophical literature on shame (Williams 1993; Nussbaum 2004) including insightful discussion of the phenomenology of shame (Zahavi 2012; Fuchs 2002). It is generally held that shame is a moral emotion; at its simplest, moral emotions are those emotions that make fundamental contributions to human morality (Prinz 2009). In this context, shame is often described in terms of its differences from guilt; whilst guilt arises in response to immoral *actions* and their consequences—such as the act of stealing from a friend—shame is centrally concerned with faults in one’s *character*(Teroni and Brunn 2011). Consequently, whilst individuals often respond to guilt through desiring to make amends or confess, feelings of shame are more likely to elicit a desire to conceal and isolate oneself.

This is not to suggest that shame should be viewed only as a disposition, removed entirely from social context. Social-interaction always risks shame, and as such, individuals often work hard to manage their social identity and avoid the shaming gaze of others (Goffman 1959). As Leeming and Boyle (2004, 383) discuss:

The embedding of emotion in the social world is more obvious for shame than for many emotional experiences, as the real or imagined presence of others seems an important feature when people say they feel ashamed. To feel ashamed has often been seen to imply not just a negative evaluation of oneself, but an awareness of this evaluation as if through the eyes of another.

Thus, whilst shame does not respond to one’s actions towards others, it does entail a belief about the judgment of others upon the self. Participants in this study describe how they fear that their bodies will be “read” by observers as indicative of morally bad eating behaviours, which in turn reveal character flaws such as greed or lack of will power. In this sense, the lived-body becomes corporeal as the shamed person imagines herself as an object in the eyes of the other (Fuchs 2002), envisioning the conclusions that will be drawn from this evaluation of her body. Hence, participants in this study imagined the judgment of others upon their bodies and eating practices and responded to this shaming gaze through concealment and isolation.

It is perhaps important to note that whilst participants described the imagined judgment of others, none relayed stories of such judgment ever being explicitly expressed towards them. This could perhaps be understood in terms of what Scambler and Hopkins (1990) call “felt stigma”. Felt stigma occurs when an individual feels shame as a result of their condition and *fears* discrimination and enacted stigma, even when such negative social repercussions never actually occur. In this way, participants like Ruth and Sarah experience “external shame” (Gilbert 1998) believing that others see one’s self as flawed and unattractive, though it is not clear that any such judgement has ever been expressed. Indeed, it is entirely possible that those around the women will see their weight gain as positive and a sign of mental or physical health; their experience of a shameful decline may correlate with what others see as improvement or progress. This observation highlights one way in which the body can be *mis*read; weight gain in the context of diagnostic crossover from AN to BN/BED—i.e. weight gain caused by binge eating—may be mistaken for recovery, and shame may prevent those experiencing diagnostic crossover from seeking help.

### Implications for Clinical Practice

The experience of shame in diagnostic crossover has implications for clinical practice. In his discussion of shame and pride, Skårderud (2007) notes that shame is a barrier to treatment, since one of the central behavioural expressions of shame is silence. Successful therapy is predicated upon an open and trusting relationship between patient and therapist, yet shame can prevent disclosure and therefore negatively affect subsequent outcome (Swan and Andrews 2003). Further, since BN and BED are less often associated with low body weight, it may be difficult to identify individuals who have experienced crossover from AN, and diagnostic crossover may be mistaken for recovery. For these reasons, it is important to find ways in which to challenge the perceived diagnostic hierarchy and so to reduce the shame and stigma associated with disorders such as BN and BED. This would hopefully make it less difficult for individuals who have experienced diagnostic crossover in the direction of AN to BN or BED to seek treatment.

One way in which to do this might be to emphasize the similarities rather than the differences between different ED diagnoses. Fairburn argues that common mechanisms are involved in the persistence of BN, AN, and other EDs, including the influence of clinical perfectionism, core low self-esteem, mood intolerance, and interpersonal difficulties (Fairburn, Cooper, and Shafran 2003). Further, AN and BN seem to aggregate in families, possibly suggesting that the two disorders are likely to have common or overlapping etiologic determinants (Strober et al. 2000). This evidence, alongside high rates of diagnostic crossover, has led some to doubt the validity of the diagnostic criteria within DSM-5 (Eddy et al. 2002). Although the present study was small with relatively few participants, the narratives collected here are rich in detail and could perhaps inform future discussion concerning the best way to classify EDs within the DSM. In such discussions, it will be important to consider both the clinical validity and utility of diagnostic categories, but also the ways in which patients interact with and value the labels that are applied to them. Whilst the experiences of patients should not decide the outcome of decisions about the classification of EDs, this paper has shown that patient experiences entail significant normative dimensions that should be heard and fairly considered, even if ultimately outweighed by other morally and clinically relevant factors.

### Avenues for Future Research

It is important to note that these findings do not necessarily apply to patients who have experienced crossover in the other direction, from BED or BN to AN. Movement “up” the diagnostic hierarchy could induce different moral emotions such as pride, and future research is needed to examine the experiences of this group. Furthermore, this was a relatively small study and involved only female participants. This may be important, since many of the moral value judgements discussed by participants are gendered and reflect a specific account of *feminine* moral virtue. Future work could examine the experiences of men and boys who have experienced diagnostic crossover, in order to fill this important gap in the literature.

## Conclusions

In this paper I have discussed the interplay between moral values and diagnostic labels in the context of EDs. For the participants in this study, crossover from AN to BN or BED was experienced as shameful, due to the bad moral character traits that the new diagnosis and behaviours were felt to represent.

Future research should explore ways that medical practitioners can identify patients experiencing shame as a result of diagnostic crossover and develop methods of support and treatment that are sensitive to that shame. A greater awareness of diagnostic crossover more generally would also help to challenge the stigma surrounding it, potentially destabilizing the diagnostic hierarchy and consequently enabling more individuals to seek help.
